# Exploring regional variability in the short-term impact of COVID-19 on property crime in Queensland, Australia

**DOI:** 10.1186/s40163-020-00136-3

**Published:** 2021-03-08

**Authors:** Jason L. Payne, Anthony Morgan, Alex R. Piquero

**Affiliations:** 1 University of Wollongong, Keiraville, Australia; 2 Australian Institute of Criminology, Canberra, Australia; 3 University of Miami, Coral Gables, USA; 4 Monash University, Melbourne, Australia

**Keywords:** COVID-19, Property crime, Queensland, Australia, ARIMA

## Abstract

Confronted by rapidly growing infection rates, hospitalizations and deaths, governments around the world have introduced stringent containment measures to help reduce the spread of COVID-19. This public health response has had an unprecedented impact on people’s daily lives which, unsurprisingly, has also had widely observed implications in terms of crime and public safety. Drawing upon theories from environmental criminology, this study examines officially recorded property crime rates between March and June 2020 as reported for the state of Queensland, Australia. We use ARIMA modeling techniques to compute 6-month-ahead forecasts of property damage, shop theft, residential burglary, fraud, and motor vehicle theft rates and then compare these forecasts (and their 95% confidence intervals) with the observed data for March through to June. We conclude that, with the exception of fraud, all property offence categories declined significantly. For some offence types (shop stealing, other theft offences, and residential burglary), the decrease commenced as early as March. For other offence types, the decline was lagged and did not occur until April or May. Non-residential burglary was the only offence type to significantly increase, which it did in March, only to then decline significantly thereafter. These trends, while broadly consistent across the state’s 77 local government areas still varied in meaningful ways and we discuss possible explanations and implications.

## Introduction

The declaration by the World Health Organization (WHO) of the novel coronavirus disease 2019 (COVID-19) outbreak, caused by severe acute respiratory syndrome coronavirus-2 (SARS-CoV-2), as a public health emergency of international concern on 30 January 2020, led governments around the world to take drastic action and implement a wide range of proactive and reactive measures to limit the spread of the disease. Collectively referred to as ’containment measures’, these strategies are designed to limit community transmission between individuals. The exact form and timing of these measures has varied between countries and, indeed, within countries, but they have generally involved some combination of travel restrictions and border controls, quarantine requirements, social isolation and distancing requirements, and the large-scale closure of various services, business and educational facilities.

The containment measures have had an immediate impact on the routine activities of individuals, fundamentally changing the way in which people move or do not move around and come together (or not) in different settings. With fewer people accessing retail outlets and business districts, and more people staying home, scholars have quickly turned their attention to the impact this might have on the routine activities which typically underscore the incidence of common property crimes. Understanding these short- and medium-term impacts is important to inform how to respond, particularly as the pandemic and various levels of containment are likely to persist for some time yet (Miller and Blumstein 2020).

To date, there has been limited exploration of the impact of the pandemic on property crime in countries beyond the global north and studies from the United States continue to dominate the empirical story (for exception see Borrion et al. 2020; Gerell et al. 2020; Halford et al. 2020; Hodgkinson and Andresen 2020; Kim and Leung 2020). Further, as we will discuss, studies have produced mixed findings, indicating that even when faced with similar changes in mobility, crime is likely to be heavily influenced by the underlying opportunity structures that feature at the local and micro-levels (Felson et al. 2020; Rosenfeld and Lopez 2020).

In this study, we use officially recorded police data from Queensland, Australia, to explore whether property crime has changed in the context of the COVID-19 pandemic. Australia is a particularly important site for international and comparative analysis because COVID-19 emerged towards the end of summer and the containment measures persisted throughout the autumn and winter months when crime rates (particularly property crime rates) normally tend towards a seasonal decline. Whereas in the northern hemisphere there is likely to be an increase in the routine activities associated with the warmer weather, in Australia the reverse is true and this has implications for how we estimate the ‘magnitude’ of the impact of COVID-19. Further, Australia is an important comparative site because of its relatively unique approach to COVID-19 and its orientation towards a strong public health perspective on containment, resulting in comparatively strict restrictions (Chang et al. 2020; Payne et al. 2020). We focus our analysis on the northern state of Queensland, in part because it is one of the only large jurisdictions to provide ready access to recorded crime data for research purposes. In addition, Queensland is Australia’s second largest jurisdiction by land area (1.85 M km^2^) and the third most populated (5.1 million residents, or roughly 3 residents/km^2^). The majority of the population lives in the south-eastern corner of the state where the capital city, Brisbane, is situated. However, it has several sizable population centers along the eastern coast and, compared to other Australian states, a greater share of its population lives in regional centers beyond the capital city. Of particular interest is that, at the time of writing, many of the local government areas which are explored in this study have still not recorded any COVID-19 cases since the pandemic began (Queensland Department of Health 2020).^1^

To this end, our study explores several common types of property crime—property damage, shop theft, residential and non-residential burglary, robbery, fraud and motor vehicle theft—and we draw upon environmental criminology to understand how these crimes might be impacted by Queensland’s containment measures. Besides being one of few undertaken outside of the United States^2^, a unique contribution of this study is that we explore regional variation in crime changes. To this end, this study of the short-term impact of COVID-19 on property crime offers a rare opportunity to explore how large-scale and rapid changes to routine activities may have influenced the volume of recorded crime.

### What restrictions have been introduced in Australia?

In Australia, containment measures to prevent the spread of COVID-19 were introduced incrementally. The entry of foreign nationals from mainland China was banned on 1 February, before further travel bans on Iran, South Korea, and Italy in early March. This was followed by self-isolation requirements on all travelers arriving in Australia introduced on 16 March. Large, non-essential, organized public gatherings of more than 500 people were also restricted from this date, as were indoor gatherings of more than 100 people. Social distancing requirements were also introduced at this time, which required individuals to maintain a distance of 1.5 m (or about 5 feet) from one another. Australian borders were closed to all non-Australian citizens and non-residents effective 20 March. The following day, the requirement that there be 4 m^2^ per person in any enclosed space was introduced. On 23 March large-scale closures of on-premise licensed premises, restaurants and cafes (except for takeaway), entertainment venues and places of worship came into effect. Further restrictions were imposed on a range of other venues, including indoor and outdoor markets, on 26 March, while limits were placed on the number of people who can attend weddings and funerals. Public gatherings were limited to two people (non-family members) from 30 March, and Australians were advised that they were only allowed to leave home for essential shopping, medical needs, exercise, or for work or education.

Queensland was the first Australian state or territory to declare a public health emergency under the *Public Health Act 2005* on 29 January, 4 days after the first Australian confirmed case, although containment measures were not introduced until the national restriction on large gatherings in mid-March. Since the non-essential business, activity and undertaking closure direction was first released on 23 March a series of revisions have been made in line with national requirements, imposing further limits on which venues and businesses may continue to operate. School closure—which have varied from state to state—came into effect on 30 March, remaining open to the children of essential service workers. Queensland borders were closed effective 26 March, with entry limited to Queensland residents, residents of border communities undertaking essential activities and other exempt persons. Non-residents were initially required to self-isolate for 14 days after crossing the border; however, as of early April—the time period for our analyses, restrictions were tightened further and only Queensland residents could cross the border. These restrictions were enforceable by law.

Towards the end of April, Queensland recorded very few daily cases and the majority were from Australian travelers returning home and residing in quarantine. On 2 May 2020, the state government announced a staged approach to re-opening the economy and the winding back containment measures. By mid-May, restaurants, pubs and bars were reopened to up to 10 patrons at any one time. On 1 June 2020, Stage 2 commenced with a full relaxation of intra-state travel restrictions and the further re-opening of business for up to 20 people.^3^

### Why is property crime likely to be impacted by COVID-19?

**Fig. 1:**
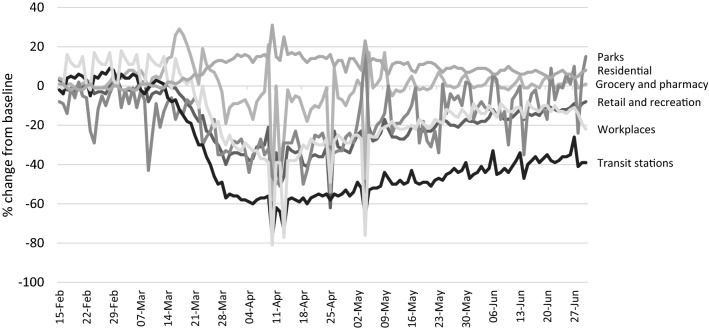
Changes in mobility over time, Queensland, 15 February–30 June. Source: Google COVID-19 Community Mobility Report

Queensland’s containment measures aimed to prevent the spread of the virus by restricting the movement of people in the community. This necessary public health measure has a profound impact on people’s everyday use of public and private space. Data collected by the Australian Bureau of Statistics (2020a) showed that, by late March, many Australians had changed their behavior and were maintaining social distance and avoiding public places, and that these behavioral changes continued well into April (Australian Bureau of Statistics 2020c). Location data reported by Google on community mobility has tracked how often and for how long people travel to different location types, compared with a baseline value (the median value for the same day of the week in January and early February). Figure 1 shows these changes over time in Queensland, and demonstrates that there have been significant reductions in visits to public spaces, including parks (down by an average of 33% over the month of April), retail and recreation premises (down 37%), workplaces (down 36%) and transit stations (down 59%). Conversely, the time spent in residential locations increased by an average of 15 percent. As happened overseas (Midoes 2020; Piquero et al. 2020), changes to mobility started prior to the implementation of formal measures. However, most of the changes were first observed in the second half of March, and were sustained through to the end of April. As shown in Fig. 1, after these changes to mobility peaked in April, and coinciding with the winding back of restrictions, levels of mobility gradually returned to values much closer to pre-pandemic levels by the end of June, with the exception of workplaces and transit stations.

The scale of these changes is such that it is plausible to expect that they will also have a measurable impact on crime, including property crime. According to Cohen and Felson’s (1979) routine activity theory, crime occurs when there is a motivated offender, a suitable target, and the absence of a capable guardian. When these converge on a more regular basis, crime is more common. Crime pattern theory combines aspects of the routine activity approach and other environmental criminological theories. It describes how offenders may come across opportunities for crime in the course of their everyday lives (Brantingham and Brantingham 1993). According to crime pattern theory, crime occurs when the activity spaces of offenders intersect with the activity spaces of a target, precipitated by some triggering event.

Research has shown that many property offenders happen across opportunities in the course of their everyday activities, rather than necessarily actively seeking out targets (Bernasco 2018; Fernandez et al. 2006). Similarly, studies have shown that offenders are more likely to commit crimes in areas where there is a large number of targets, and these targets are easily accessible (Ruiter 2017; Townsley et al. 2015). Obviously, major disruptions of these routine activities should have a significant impact on when, and where, we might expect crime to occur.

It is possible to outline a number of hypotheses about the expected impact on property crime. In practice, the impact is likely to vary by both crime type and by setting. Limiting the amount of time that people are permitted to spend outside of the home, and the number of people outside the home with whom people may have contact, reduces opportunities for interactions between potential victims (or targets) and motivated offenders in public places. This is particularly relevant to those crime types that might often occur largely as a consequence of these interactions, particularly theft offences. Consider, for example, the would-be offender who happens across an unattended bag in a train station, or unattended vehicles parked in an open-air car park.

Then there is the closure of certain premises, most notably entertainment venues, restaurants and cafes, both as a direct requirement of government policies, or as an indirect consequence of the economic impacts of social distancing (e.g., stores that have closed because customer numbers are down or because the store cannot safely meet the social distancing requirements). This might be expected to significantly impact those offences that occur in and around these settings; namely, shop stealing offences. In those stores that continue to operate, there has been a notable increase in security and retail staff to manage compliance with social distancing and ensure shelves are restocked, respectively. These may act as place managers and deter offenders who target these open stores.

At the same time, in some locations—primarily residential settings—there will be a marked increase in guardianship because people are confined to their homes. We know that the presence of guardianship has an important influence of offenders’ decisions to target certain properties. Normally, this guardianship varies considerably over the course of the day, which shapes when crime is most likely to occur (Reynald and Elffers 2015). Residential burglary, and other property crimes in residential settings, including vehicle theft and theft from vehicles, may be expected to decline. Conversely, commercial premises are likely to become more susceptible to crime, given the fall in guardianship that coincides with the closures of businesses.

There are other crime types for which the impact is likely to be far more dependent on the unique characteristics of sub-categories of offences. For example, in the case of property damage, alcohol-related property damage that occurs within the context of the night time economy may decrease, while graffiti and vandalism in public spaces may increase due to reduced surveillance and increased opportunity (less time spent at work or in school). Likewise, there has been growing concern about the impact of COVID-19 on fraud offending, as profit-motivated offenders look to exploit opportunities in weakly controlled financial systems, and the fear and anxiety that might make individuals susceptible to scams (Australian Cyber Security Centre 2020). Conversely, offences involving ‘tap and go’ and online fraud using stolen credit cards may become more difficult, as offenders are no longer able to steal these cards to commit a fraud offence, or then (in the case of ‘tap and go’ fraud) have limited options in terms of stores to target (for the reasons explained above).

Of course, crime is not evenly distributed across place or time. Brantingham and Brantingham (1995) also identified the significance of crime attractors and crime generators. Crime generators are those places that attract large numbers of people, which provide opportunities for targets and offenders to come together in space and time. Shopping centers and public transport are both examples of crime generators (Newton 2018). Crime attractors are places that are attractive to motivated offenders because they provide opportunities for crime to occur. Examples include certain licensed premises in the night time economy, poorly secured car parks and drug markets. Because of their crime generating and crime attracting properties, these are common hotspots, including for property crime. Many of these location types have all been profoundly impacted by containment measures. Because they are crime hotspots, these changes to routine activities might be expected to have a disproportionate impact in terms of their share of any crime reduction effects.

There is also strong evidence, including from Queensland, that crime is not evenly distributed across communities (Allard et al. 2012; Wickes et al. 2017). This too is likely to be a function of both the opportunity structures in these communities (e.g., levels of guardianship), and the underlying social conditions that influence the propensity of people living in these communities to commit crime. Research into the crime drop has shown that crime declines over the past two decades have not been evenly distributed (Hodgkinson and Andresen 2019). It is reasonable then to expect that the impact of containment measures will also vary and is consistent with the research on crime at micro places (Weisburd et al. 2004).

Further, it is true that crime will not simply stop completely. While there is overwhelming evidence of the impact of guardianship on property offending, particularly burglary, the presence of informal guardians is not sufficient to deter all offenders all of the time (Reynald 2017). In addition, not everyone complies with social distancing measures. There is some overlap between the profile of those people who are less likely to comply and those people who are more likely to be involved in crime. Specifically, in a study of social distancing behavior among young people, Nivette et al. (2020) found that non-compliance was associated with an increased propensity for delinquent behavior and association with delinquent peers. It is plausible that these non-compliant individuals will be more likely to offend, reducing any effect from the changes to routine activities of compliant individuals. There is also the genuine probability of crime displacement. For example, a decrease in residential burglary because of increased guardianship will likely coincide with an increase in commercial burglary for exactly the opposite reasons. However, we know from a large body of displacement literature that this is unlikely to offset reductions in crime, at least not entirely (Guerette and Bowers 2009).

Further, it is possible that changes to routine activities become less influential—particularly as containment measures are relaxed—but that the pandemic will lead to an increase in motivated offenders. There is already evidence of the negative psychological impacts of quarantine measures and the other financial consequences resulting from the economic impacts of COVID-19. Levels of stress and depression are much higher than usually observed, particularly among individuals with a pre-existing mood disorder (Australian Bureau of Statistics 2020b; Van Rheenen et al. 2020). Further, one in three Australian households already report being financially worse off (Australian Bureau of Statistics 2020b). In fact, the number of people employed in Australia fell by nearly 600,000 people in April alone (Australian Bureau of Statistics 2020d). Unemployment, under-employment, and the strain associated with the significant and mounting financial pressures may exert an upward effect on property crime by increasing the negative emotions (anger, anxiety, desperation) that are conducive to antisocial behavioral adaptations (Agnew 1992).

In Agnew’s original description of the link between strain and crime, it is clear that his focus was predominantly on the longer-term unfolding of strain-induced adaptive behaviors. Indeed, a strict reading of Agnew’s earlier texts might lead one to conclude that any strain caused by COVID-19 is unlikely to feature as an explanation for short-term changes in crime rates. We generally agree with this position and believe that the longer-term impact of COVID-19 will unfold as the pressure and strain of the pandemic persists and as strains become more frequent and of long-duration, two characteristics related to strain that Agnew highlighted as being crime-exacerbating. That said, we also believe that there is room for strain in the exploration of short-term changes in crime. Specifically, the extra and more acute pressure that COVID-19 has likely added to people’s lives cannot be discounted, especially its impact on those who, before the pandemic, were already experiencing strain-related negative emotions of long-duration and high-frequency. Perhaps the strains associated with the pandemic might not have been as salient in triggering the onset of offending among the vast majority of the population who are not already engaged in crime (though this is possible for some individuals who may have lost their job and needed to turn to crime to obtain economic resources to survive), but it could most certainly exacerbate and make more acute the strains experienced by those who were already engaged in antisocial adaptations.^4^

### What trends have been observed around the world?

Despite the significant disruption associated with containment measures, analyses of the crime impact of COVID-19—of which there are a growing number—have tended to find smaller than expected reductions in property crimes. Findings from these studies have also been notable for the variability in terms of percent reductions as well as for which types of property crime have been impacted (see e.g., Boman and Gallupe 2020).

Mohler et al. (2020) analyzed daily counts of calls for service and recorded crime in Los Angeles and Indianapolis. They compared the period after stay at home orders had been issued (March 20 and March 24, respectively)—when full social distancing came into effect—to the period prior to school closures. In terms of property crime, there were significant declines in burglary and robbery calls for service in Los Angeles, and a small increase in vehicle theft, but no change in Indianapolis. However, even where differences in call volumes were observed, they were relatively small in practical terms. Similar trends were observed in recorded crime, but only robbery in Los Angeles was significantly reduced. These findings were reinforced in a second regression that incorporated Google mobility data.

Campedelli et al. (2020a) analyzed daily recorded crime counts over a three-year period in Los Angeles using Bayesian structural time-series models to produce a synthetic counterfactual—what would have occurred had there been no containment measures—which they compared with observed crime counts during a period of increasingly stringent social distancing measures in March. Property crimes, including shoplifting and thefts, decreased, as did crime overall, and these trends were amplified during the period of more stringent measures. In a follow-up paper, Campedelli et al. (2020b) assessed how COVID-19-related containment policies were associated with different crime types at the community-level in Chicago and found that changes in crime trends differed across both communities and crime types.

Ashby (2020a) forecasted the expected frequency of crime during the pandemic in 16 large US cities based on data from 2016 to early 2020 using seasonal auto-regressive integrated moving average models to produce the synthetic counterfactual. There was no change in crime levels between the observed and predicted values before early March, when social distancing measures (like closing schools and then stay at home orders) were introduced. There was some evidence of an impact on property crime, but this varied between cities and over time: residential burglary decreased for two consecutive weeks or more in three cities, non-residential burglary increased in one city, and theft from vehicles increased in some cities and decreased in others; however, no two cities exhibited the same trends in crime. Ashby (2020b) repeated this analysis with calls for service data in ten large cities, finding significant reductions in calls to intruder alarms in four of them.

In analysis of Detroit burglaries in March 2020, Felson and his colleagues (2020) separated 879 blocks grouped by the type of land use, i.e., residential versus more mixed. Then, after parceling the monthly data into three periods (pre-containment, transition period, and post-containment) the authors found that burglaries increased in block groups with mixed land use but did not so in the residential comparison areas.

Finally, Rosenfeld and Lopez (2020) examined eleven crime rates for eleven different offence types in 27 US cities during the COVID-19 period. While there was variability in data access for all offence types across all cities, generally speaking the results showed that most crime types decreased, with residential burglaries, larcenies, and drug offences failing significantly during the pandemic, while homicides and aggravated assaults rose significantly in late May and June 2020.

Relatively few studies have been undertaken to explore the impact of COVID-19 containment measures on property crime outside of the United States. The first examined crime in Sweden, where containment measures have been less stringent than elsewhere. Gerell et al. (2020) compared the weekly crime numbers in 2020 with the median from three previous years, and observed declines in residential burglary, commercial burglary, and pickpocketing. The decline in pickpocketing offences was particularly noteworthy. Hodgkinson and Andresen (2020) analyzed crime levels in Vancouver, Canada, observing an initial increase, then decrease, in commercial burglary, while theft and stealing from motor vehicles also declined. Motor vehicle theft was stable at a time when it would normally be increasing. There was no immediate impact on residential burglary.

Halford et al. (2020) examined changes in recorded crime in the United Kingdom, using similar ARIMA forecasting methods to other studies and comparing actual crime levels against the synthetic control. They observed significant declines in major categories of property crime, including shoplifting, criminal damage, theft from vehicle and burglary (dwelling and non-dwelling), although daily rates of burglary remained within the confidence intervals for each day in the forecast. They also computed the mobility elasticity of crime for three property crime types—shoplifting, residential burglary and theft from vehicle—which was 2.0, -1.0 and 0.7, respectively. Meaning, that the decrease in shoplifting was twice the decrease in time spent in retail areas, burglary decreased by the same proportion as time spent in homes increased, and the decline in theft was less than the relative decline in time spent at work (used as a proxy for travel time).

In China, Borrion et al. (2020) assessed changes in commercial theft and found a large reduction (64%) during an 83-day period, which then returned to their previous-COVID-19 levels and, in some cases, even increased.

Significant statewide crime declines have also been observed in Australian studies. Kim and Leung (2020) used five different forecasting methods to forecast several property crime types between mid-March and late April, finding differences between forecasted and observed incident counts in the range of 24 to 34 percent for residential and non-residential burglary, motor vehicle theft and stealing from motor vehicles. Robbery was down by 42 percent, while the observed rate of steal from retail stores was less than half the forecasted levels. More recently, Rmandic et al. (2020) compared offence numbers for the period April to June 2020 (prior to the second wave of COVID-19 infections which impacted Victoria in the second half of 2020) with the same period in 2019, reporting declines of 13 percent for steal from a motor vehicle, 28 percent for stealing from retail stories and 27 percent for residential non-aggravated burglary. Although they limited their analysis to overall offence numbers, they also found considerable variability in the size and direction of changes in recorded crime at the Local Government Area (LGA) level.

### Current focus

Overall, the strongest evidence in terms of the short-term impact of COVID-19 appears to be for property crimes in locations directly impacted by containment measures—particularly retail settings—and those which are likely to be the result of the incidental contact between motivated offenders and suitable targets/victims in public places (i.e., theft). There has been mixed evidence with respect to burglary, despite the significant increase in guardianship in the home, with some evidence of declines in residential burglaries but increases in non-residential burglary. Further, the variation between cities suggests different effects, which is likely a consequence of the different containment measures (and timing of those measures) and different patterns of crime that exists within those settings; the latter being heavily influenced by underlying opportunity structures. This is reflected in results of the small number of studies which have disaggregated results by neighbourhood or LGA (Campedelli et al. 2020b; Rmandic et al. 2020). Our study aims to add to this knowledge by exploring trends in property crime amidst the social distancing measures put into place to deal with the pandemic in Queensland Australia. An additional feature of our analysis is the exploration of potential variability of specific crime types at smaller units of analyses—aside from the state more generally. Our hypothesis is that property crime rates in Queensland will have declined in the context of the social distancing and containment measures that were introduced. Further, we expect these declines will have been greatest for those property crime types (and in those areas) where routine activities and opportunity structures are most influential (retail, other theft and burglary).

## Methodology

### Data

These data are drawn from the Queensland Government’s Open Data Portal (ODP; Queensland Government 2020). The ODP reports statewide monthly offence rates per 100,000 of the Queensland population. It also reports the offence rates for each of the 77 individual local government areas (LGAs) in Queensland. For this study, we use the offence rates for five types of property crime—property damage, shop theft, burglary, fraud (including the subcategory of credit-card fraud), and motor-vehicle theft.^5^

### Analytical approach

For each of the property offence categories, we operationalize an Auto Regressive Integrated Moving Average (ARIMA) model on the monthly offence rate between February 2014 and February 2020.^6^ To do this, we use *fable* v0.2.1 (O’Hara-Wild et al. 2020), a time series forecasting routine operationalized for use with the statistical program R. ARIMA models are a specific type of time series forecasting technique which capitalize on several key time-series parameters–the series trend and seasonality, its lagged auto correlations and its lagged partial auto-correlations (correlations between residual errors).^7^ These patterns are then used to specify a statistical model from which forecasts can be computed. Like all models, the relative accuracy of any forecast depends on the strength of the relationship between past and future values.

Using *fable*, the final ARIMA specification for each property crime type is derived from an iterative parameter search algorithm. This is not driven by any specific theoretical model of the predictive pattern of *AR* and *MA* parameters, but instead, it is a data-driven model-selection process that identifies the model of best fit given the available data. The search algorithm is a variation of that which was proposed by Hyndman and Khandakar (2008) for automatic ARIMA modelling. Specifically, the need for trend and seasonal differencing is first identified using a series of KPSS tests (see Kwiatkowski et al. 1992). *Fable* automates the KPSS test and performs an OLS regression with terms for deterministic trend, random walk, and stationary error. Whether the ARIMA specification requires trend and seasonal adjustment depends on whether the relevant parameters meet the necessary alpha threshold of (p < 0.05). Once adjusted, the *AR* and *MA* parameters are chosen through a series of comparative model analyses using four baseline^8^ models which are calculated and compared. The best of these baseline models (based on the model which produces the smallest Aikake Information Criteria (AIC)) is then used as the starting point for an iterative model search function that tests single unit changes to the *AR* and *MA* parameters. Any new model that outperforms the starting model (again, based on it producing a smaller AIC) is selected and the iterative search function is repeated until such time as no alternative model can be found (see Hyndman and Athanasopoulos 2020).

At the conclusion of the automated ARIMA modelling procedure, two estimates of model accuracy are calculated—the Mean Absolute Scaled Error (MASE) and the Mean Absolute Percentage Error (MAPE). Both are derived by ‘training’ each model on the in-sample data for 2014–2018 and then comparing the model performance against the out-sample data for 2019. The MASE (see Hyndman and Koehler 2006) is a numerical representation of the error produced by the forecast for 2019, compared to the average error produced by a naïve model for 2014–2018. A MASE estimate less than the value of one implies that the ARIMA model out-performs a basic one-step ahead naïve model. A value greater than one implies the model is no better (or possibly worse) than a naïve estimation. MAPE is an estimate of the average error produced by the forecast in for the out-sample data in 2019. In this case, month-by-month error is defined as the percentage difference between the forecast and the observed offence rates. The average of these error percentages represents the MAPE. Crucially, offence series with little or no offence counts will produce extreme estimates of MASE greater than the value of one (values approaching infinity). Similarly, some offence counts are simply too low or longitudinally volatile to produce reasonable ARIMA forecasts. These models result in large values for MAPE. In this study, we treat any model with extreme values of MASE (> 100) and/or MAPE (> 50%) as ‘not able to be estimated’.

For all estimable models we then use the final specification to forecast point estimates and 95% confidence intervals for March through to August 2020. We then compare the observed offence rates for those months to the forecasted point estimate and consider its position within the confidence interval. We only conclude that the offence rate has changed under COVID-19 conditions if the observed value (for March to June 2020) falls outside the upper or lower bounds of the 95% confidence interval.

Finally, to explore geographical heterogeneity we repeat the procedures above on the offence-specific time-series data for each of the 77 LGAs in Queensland. A total of 624 models were executed and the results are summarized here, with a specific focus on the 12 LGAs where the population is greater than 100,000 (Table 1). The final model specifications and output of this analysis can be found in the supplementary tables associated with this manuscript.^9^

**Table 1:** Summary of ARIMA modelling outcomes

	Estimated resident population	Theft and related offences												Property damage			
		Shop stealing				Other stealing				Motor vehicle theft				All property damage			
		March	April	May	June	March	April	May	June	March	April	May	June	March	April	May	June
State-wide summary (of 77 LGAs)																	
Estimable models (a)	−	24				38				22				41			
LGAs with increase (n significant) (b)	−	4 (1)	4 (0)	2 (0)	1 (0)	15 (1)	1 (0)	2 (0)	1 (0)	13 (4)	3 (0)	3 (0)	5 (0)	20 (2)	6 (0)	6 (0)	8 (0)
LGAs with decrease (n significant) (c)	−	20 (3)	20 (14)	22 (14)	23 (11)	23 (4)	37 (32)	36 (24)	37 (20)	9 (0)	19 (11)	19 (5)	17 (9)	21 (1)	35 (10)	35 (10)	33 (3)
Key LGA Snapshot (% difference from forecast) (d)																	
Brisbane City Council	1253982	***−11.5***	***−46.7***	***−37.9***	***−31.2***	−4.0	***−43.0***	***−48.4***	***−44.1***	11.5	***−26.1***	***−31.8***	***−34.6***	−4.5	***−21.0***	***−17.6***	−15.3
Gold Coast City Council	620518	−1.7	***−52.8***	***−30.0***	***−36.7***	−12.4	***−46.6***	***−43.3***	***−45.0***	***32.1***	−3.3	−18.2	***−38.2***	−0.4	***−19.3***	***−30.3***	−7.3
Moreton Bay Regional Council	469465	−20.2	***−63.3***	***−50.1***	***−46.0***	***−15.7***	***−40.1***	***−54.5***	***−56.0***	3.1	***−27.7***	−20.5	***−38.5***	−13.5	***−30.7***	***−27.1***	***−25.1***
Logan City Council	334358	9.8	***−46.1***	−23.5	***−33.9***	***−16.4***	***−43.8***	***−62.0***	***−57.0***	2.2	***−27.8***	***−36.1***	***−32.0***	***25.4***	−15.7	−9.5	***−24.1***
Sunshine Coast Regional Council	328428	−10.7	***−65.7***	***−45.9***	***−45.9***	*−4.9*	***−38.3***	***−42.2***	***−40.0***	***23.7***	−11.9	−10.1	−17.5	-9.7	***−34.7***	−22.7	−17.8
Ipswich City Council	222307	−12.8	***−51.2***	***−60.3***	−28.4	*−15.5*	***−48.6***	***−63.7***	***−58.6***	−16.3	***−46.1***	−29.0	***−39.0***	−13.9	−23.5	***−42.8***	−18.7
Townsville City Council	195032	***−41.5***	***−70.9***	***−49.3***	***−71.9***	*−17.4*	***−55.8***	***−64.0***	***−62.8***	N.R.E				***−31.1***	−23.8	***−32.9***	−24.5
Toowoomba Regional Council	169008	−9.5	−40.7	***−44.8***	−26.8	*−13.8*	−35.5	−32.6	−21.7	***20.9***	−35.4	−9.5	−12.4	21.3	0.6	−19.5	0.5
Cairns Regional Council	166862	−13.5	***−56.1***	***−53.8***	***−59.3***	*13.3*	***−43.5***	***−59.6***	***−43.3***	***-25.9***	***−49.8***	−43.8	−37.2	−14.5	***−28.6***	−15.4	−8.4
Redland City Council	158815	−6.4	−6.4	−15.3	−30.9	*−10.6*	***−34.7***	***−48.2***	***−47.0***	***-6.5***	***−51.4***	−36.4	***−47.7***	9.7	−18.1	−28.3	−35.6
Mackay Regional Council	116763	−28.7	***−50.6***	***−70.8***	−36.5	*−12.1*	***−46.7***	***−49.4***	***−45.4***	***5.0***	−28.5	***−44.1***	***−44.1***	33.6	1.6	−8.5	−21.6
Fraser Coast Regional Council	106712	−13.9	−31.5	−40.4	***−66.9***	*−4.3*	***−38.4***	***−44.0***	−28.1	***25.5***	***−53.6***	−17.5	3.1	***35.4***	6.8	16.5	−23.1
* State-wide*	*5130000*	**−*13.3***	**−*49.0***	**−*44.9***	**−*42.3***	**−*10.5***	**−*42.7***	**−*51.5***	**−*45.3***	−*1.7*	**−*37.7***	**−*40.9***	**−*41.6***	−*4.4*	**−*24.2***	**−*21.8***	**−*12.4***

	Estimated Resident Population	Burglary								Fraud				Robbery			
		Residential				Other				All fraud				Armed and unarmed			
		March	April	May	June	March	April	May	June	March	April	May	June	March	April	May	June
State-wide summary (of 77 LGAs)																	
Estimable models (a)	–	23				25				13				8			
LGAs with increase (n significant) (b)	-–	15 (3)	1 (0)	0 (0)	4 (1)	21 (7)	7 (0)	4 (0)	2 (0)	2 (0)	1 (0)	1 (0)	1 (1)	3 (0)	0 (0)	0 (0)	0 (0)
LGAs with decrease (n significant) (c)	-–	8 (2)	22 (19)	23 (9)	19 (8)	4 (0)	18 (8)	21 (3)	23 (5)	11 (0)	12 (0)	12 (0)	12 (0)	5 (0)	8 (6)	8 (3)	8 (4)
Key LGA Snapshot (% difference from forecast) (d)																	
Brisbane City Council	1253982	−12.4	***−49.7***	***−49.9***	***−49.1***	11.7	***−20.0***	***−27.2***	***−44.5***	***−11.7***	***−24.8***	***41.6***	***−20.5***	25.9	***−48.5***	−28.8	***−43.2***
Gold Coast City Council	620518	***50.9***	0.5	***−39.7***	***−33.8***	***54.9***	27.5	9.8	−8.0	***−24.6***	***−8.3***	***−38.3***	***−18.9***	−14.3	−28.6	−32.2	***−53.6***
Moreton Bay Regional Council	469465	−5.7	−19.6	***−37.5***	***−37.0***	25.9	−21.2	−16.1	−29.5	***−31.5***	***−50.8***	***−29.4***	***−29.0***	−23.2	−28.4	−48.8	−23.2
Logan City Council	334358	5.0	−23.5	***−51.9***	***−38.4***	***26.1***	−3.4	−9.2	−17.1	***−7.9***	***−14.1***	***−17.7***	***−3.8***	−13.8	***−61.2***	−22.4	−0.9
Sunshine Coast Regional Council	328428	17.4	***−47.1***	−43.3	−26.1	42.1	24.1	−19.7	3.0	−32.4	−20.3	-8.2	−43.5	N.R.E			
Ipswich City Council	222307	−9.4	***−44.2***	−35.1	−37.7	8.1	***−45.9***	−33.2	***−55.8***	−27.1	−6.4	−24.4	−43.3	−14.9	−53.6	***−69.1***	−38.1
Townsville City Council	195032	N.R.E				21.6	***−44.3***	***−52.7***	***−61.2***	98.1	−65.0	−44.4	-2.2	N.R.E			
Toowoomba Regional Council	169008	6.0	***−56.3***	***−55.5***	***−35.0***	-1.1	−19.7	−19.7	-4.5	−47.6	38.5	−59.2	***163.5***	N.R.E			
Cairns Regional Council	166862	***−37.6***	***−48.8***	***−56.8***	−46.7	21.6	−4.0	−37.8	***−64.4***	−30.0	−15.4	−18.4	−26.1	39.0	−12.2	***−78.1***	−12.2
Redland City Council	158815	***59.7***	−34.2	−6.1	−21.7	***77.3***	23.6	−43.8	−43.8	0.2	−2.3	−61.0	−30.7	N.R.E			
Mackay Regional Council	116763	22.2	−23.0	−53.2	−30.8	3.7	−10.2	−70.3	−52.2	N.R.E				N.R.E			
Fraser Coast Regional Council	106712	***104.8***	−23.0	−32.2	−7.2	10.8	−0.3	−37.2	−40.9	N.R.E				N.R.E			
* State-wide*	*5130000*	−*11.5*	**−*50.4***	**−*58.6***	**−*54.2***	***23.1***	**−*16.6***	**−*31.7***	**−*40.9***	−*19.4*	−*30.5*	−*11.2*	−*21.0*	*2.2*	**−*41.4***	**−*41.0***	**−*32.5***

Source: Queensland Government Open Data Portal

Bold italic values indicate increases or decreases that were statistically significant at *p* < 0.05

^a^The number of LGA-level ARIMA models (of 77 LGAs) which satisfied the robust estimation requirements for inclusion

^b^The number of estimable LGA-level models where the observed crime rate was higher than the forecasted rate in each month. The value in parentheses indicates the number of models where the increase was statistically significant

^c^The number of estimable LGA-level models where the observed crime rate was lower than the forecasted rate in each month. The value in parentheses indicates the number of models where the decrease was statistically significant

^d^The relative difference between the observed rate and the forecasted rate is measured as a percentage increase or decrease from the predicted point estimate

## Results

### Theft and related offences

**Fig. 2:**
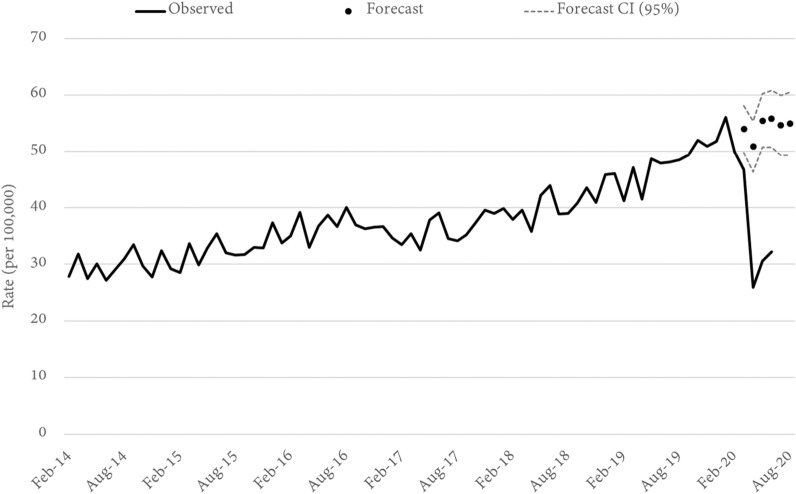
Queensland SHOP STEALING rates and forecasts. Source: Queensland offence rates, Open Data Portal

**Fig. 3:**
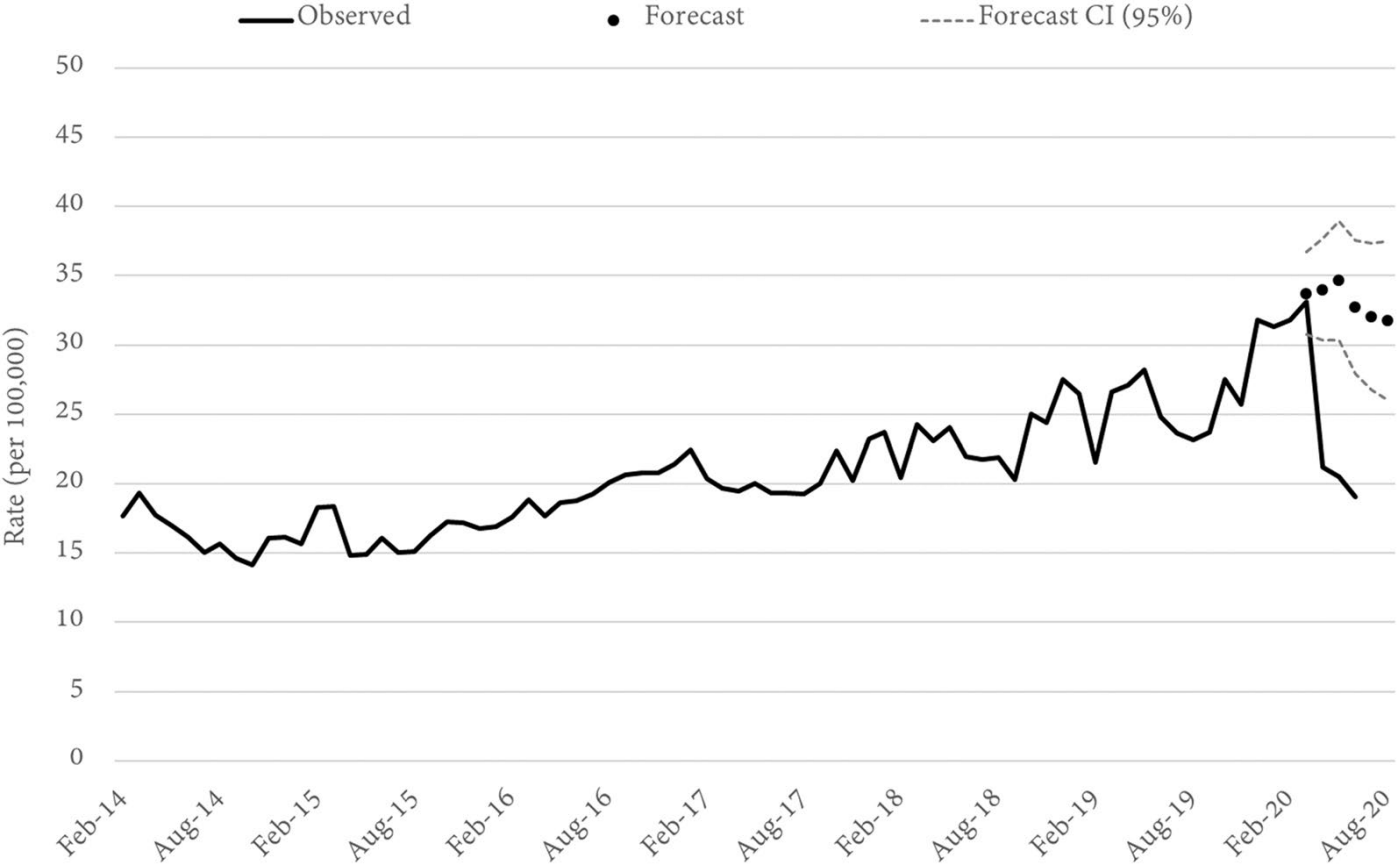
Queensland MOTOR VEHICLE THEFT rates and forecasts. Source: Queensland offence rates, Open Data Portal

**Fig. 4:**
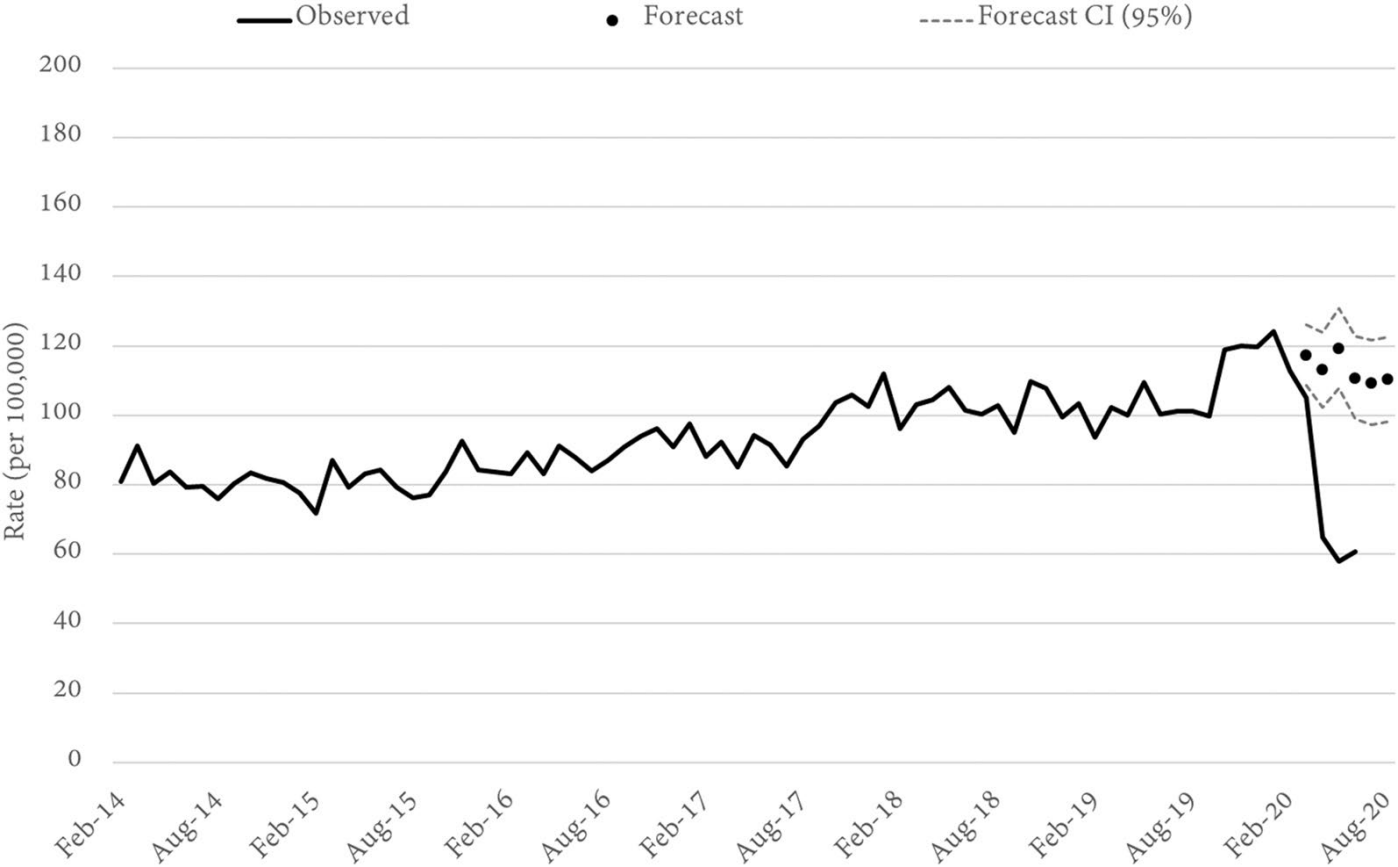
Queensland OTHER THEFT rates and forecasts. Source: Queensland offence rates, Open Data Portal

Three alternative theft offences were examined. These were shop stealing and retail theft, motor vehicle theft, and other theft (not elsewhere classified). In all three cases the statewide rate of offending for April, May, and June (per 100,000) was significantly lower than forecast. In April, for example, shop stealing was 49 percent lower than forecast (Fig. 2), while motor vehicle (Fig. 3) and other (Fig. 4) theft were lower by 38 and 43 percent, respectively. Notably, both shop stealing and other theft were significantly lower than forecast in March (when mobility began to change but the main government restrictions were yet to be implemented), while motor vehicle theft remained on-trend until a sharp fall in April. It is also notable that the largest relative decrease in each of the three theft offences occurred in different months. The decline in shop-stealing, for example, was largest in April, after which the offence rate trended up again, although still well below forecast. For other theft offences, the lowest rate was recorded in May (down 52%), while the lowest recorded rate of motor vehicle theft was in June (down 42%).

Geographically, the results paint a mixed picture (Table 1). In terms of shop-stealing, 24 models (of 77 LGAs) could be reliably estimated from their historical series. Of these, the recorded offence rate in April was lower than forecast in 20 LGAs, 14 of which were statistically significant. Only 4 LGAs recorded a shop-stealing offence rate that was above the forecast for April, but in none of these four instances did the estimate exceed the confidence interval. For motor-vehicle theft, models were produced for 22 LGAs and by April, 19 had recorded an offence rate lower than forecast (11 of these declines were statistically significant). In terms of other-theft offences. 38 LGAs were modelled and 37 recorded a lower-than-forecast result in April. Of these, the decline was statistically significant in 32 LGAs.

Focusing specifically on the 12 LGAs with a population greater than 100,000 we note a number of interesting results. The first is the overwhelming consistency in trend, with all 12 LGAs trending downwards for all three theft offence types by April. Not all of these declines were statistically significant, but the trend was universal. Within this general story of homogeneity are a series of notable differences. For example, while the statewide data showed the largest decline in shop-theft occurring in April, this was not the universal experience of all LGAs. The lowest rate in Redlands City Council and Fraser Coast Regional Council was, in fact, seen in June (down 31 and 67% respectively), while the declines in April and May were comparatively modest. In Redlands, none of the declines in shop stealing were statistically significant while in Fraser Coast, only the June figure exceeded the 95 percent confidence interval. For other theft offences, Toowoomba Regional Council appears as an outlier having only relatively modest declines that were not statistically significant. Motor vehicle theft in the Gold Coast City Council region (a southern residential and tourist location) is also a notable outlier for having a statistically significant increase (+ 32%) in motor vehicle theft in March, and only a modest (non-significant) decrease in April (−6%).

### Burglary

**Fig. 5:**
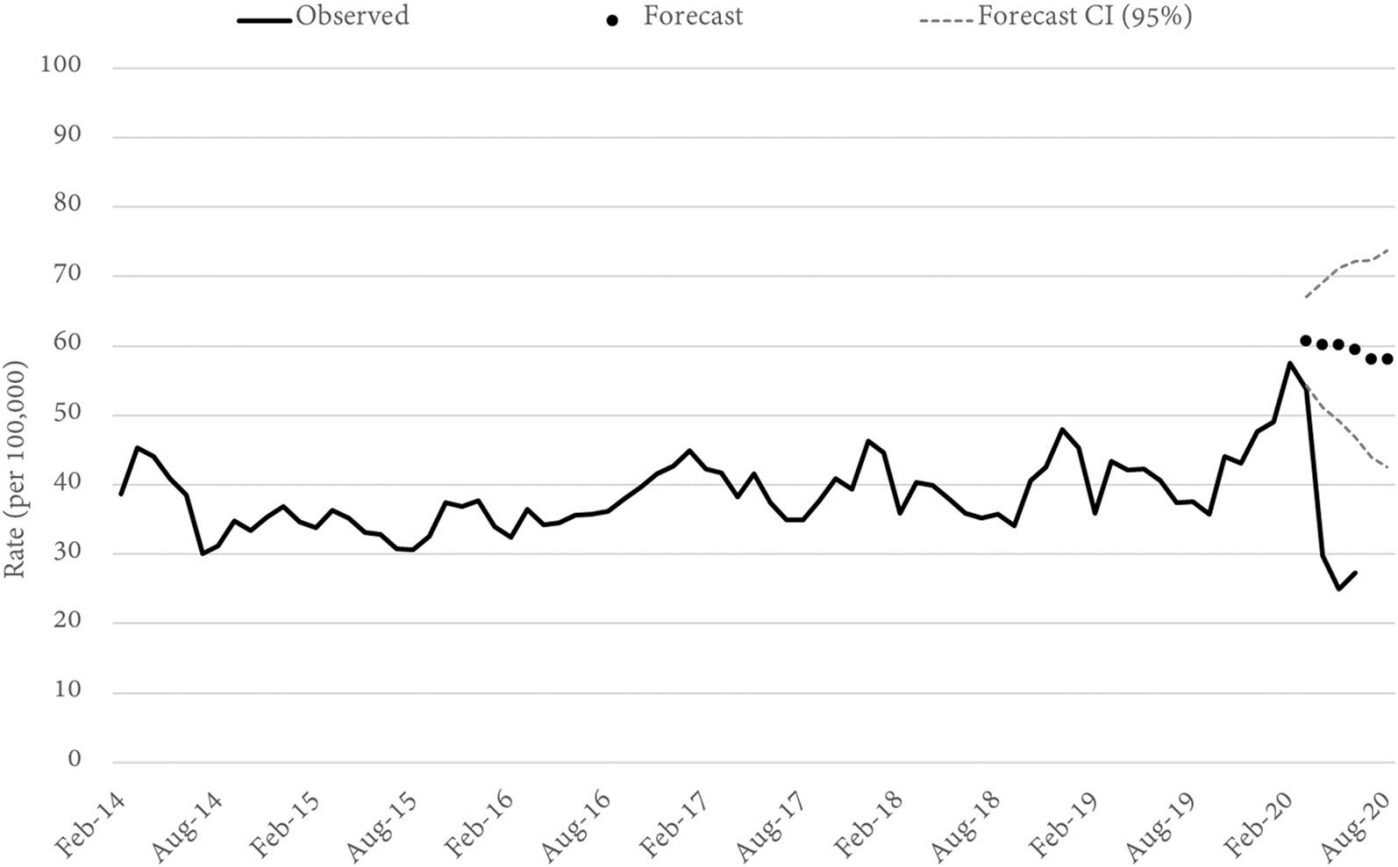
Queensland RESIDENTIAL BURGLARY rates and forecasts. Source: Queensland offence rates, Open Data Portal

**Fig. 6:**
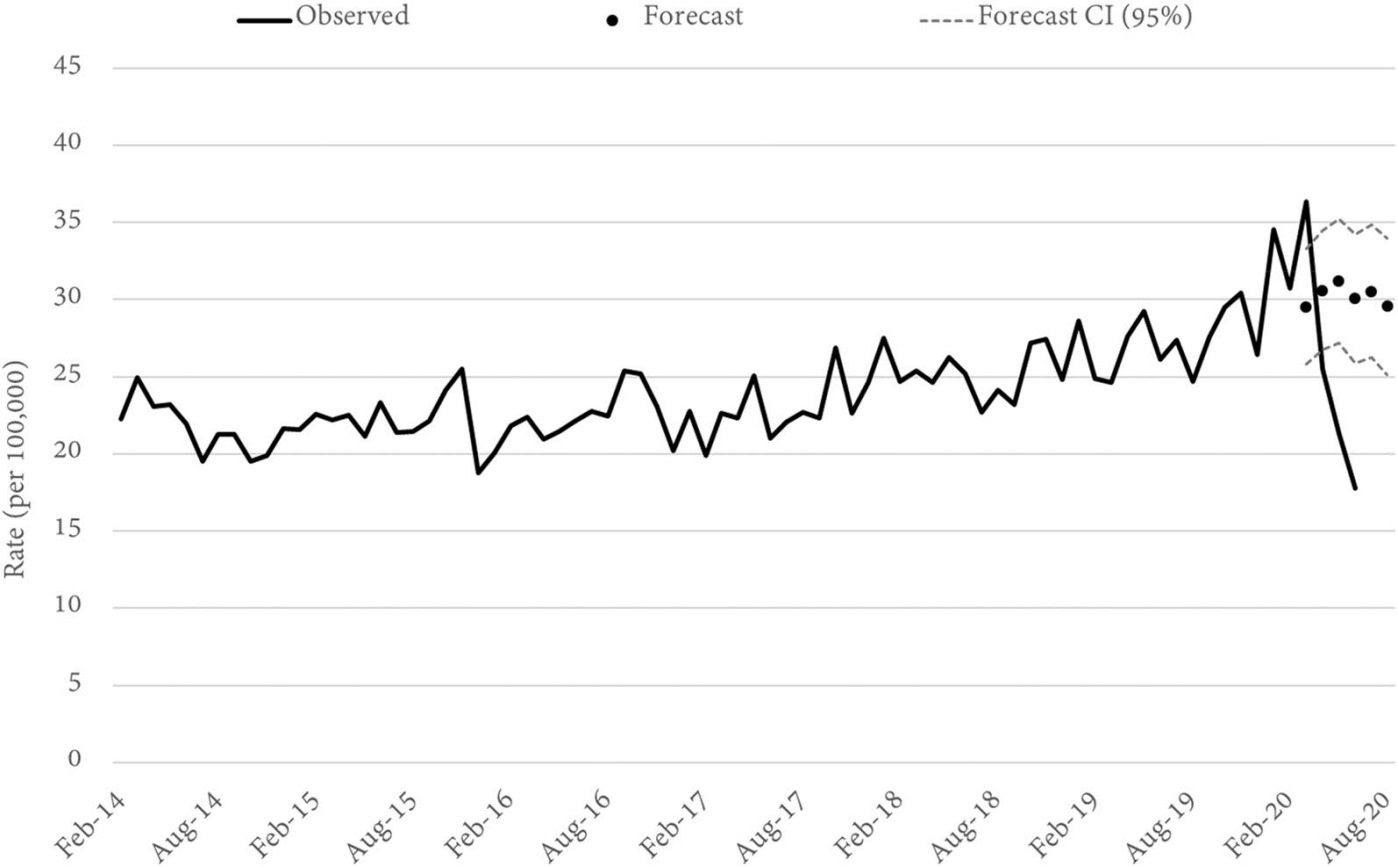
Queensland NON-RESIDENTIAL BURGLARY rates and forecasts. Source: Queensland offence rates, Open Data Portal

Both residential and non-residential burglary were examined and, across the state of Queensland, both were significantly lower than forecast in April, May, and June. Residential burglary was 50 percent lower than forecast (Fig. 5) while non-residential burglary (Fig. 6) was 17 percent lower. Unlike retail and other theft related offences, burglary rates did not begin to decline in March as mobility began to shift but before formal restrictions were put into place. Statewide, residential burglary was down in March, but not significantly, while non-residential burglary was actually up by 23 percent (a statistically significant increase). We note that non-residential burglary was the only property offence type to experience a significant increase in March.

Across the state, residential burglary trends were able to be modelled for 23 LGAs. Of these, 22 LGAs recorded a lower-than-forecast decline in April and the downward shift was statistically significant in 19 LGAs. After April, rates of residential burglary remained below trend in all LGAs (at least where models could be estimated), but a fewer number were statistically significant (n = 9). In June, the downward trend had reversed in at least four LGAs and for one LGA the residential burglary rate in June was, in fact, statistically higher than forecast. The reverse in trend appears to have occurred in the less populated regional LGAs to the west and north of the capital city region.

For non-residential burglary, 25 models could be estimated. Consistent with the trends mentioned above, the data for March show that non-residential burglary rates were higher than forecast in a majority of LGAs (n = 21, statistically significant in 7). By April, the trend had reversed in most LGAs (n = 18), and was statistically lower for eight. In all, the trends in non-residential burglary from March to June were quite variable across the state (Table 1). Despite this, there are a number of notable patterns across the most populous LGAs. First, non-residential burglary rates in March were significantly higher in the Gold Coast, Logan City, Sunshine Coast and Redland City Council. All four are outer-city suburban areas with large tourist or industrial economies. In none of these four locations did the rate of non-residential burglary then decline in April, May, or June to a point where the rate was statistically lower than forecast. Second, only three LGAs experienced large, consistent and statistically significant declines from April through to June. These were Brisbane, Ipswich and Townsville LGAs. Brisbane is the inner-city metropolitan region with many retail businesses and restaurants, while Ipswich (to the west) and Townsville (to the north) are regional cities.

### Fraud

**Fig. 7:**
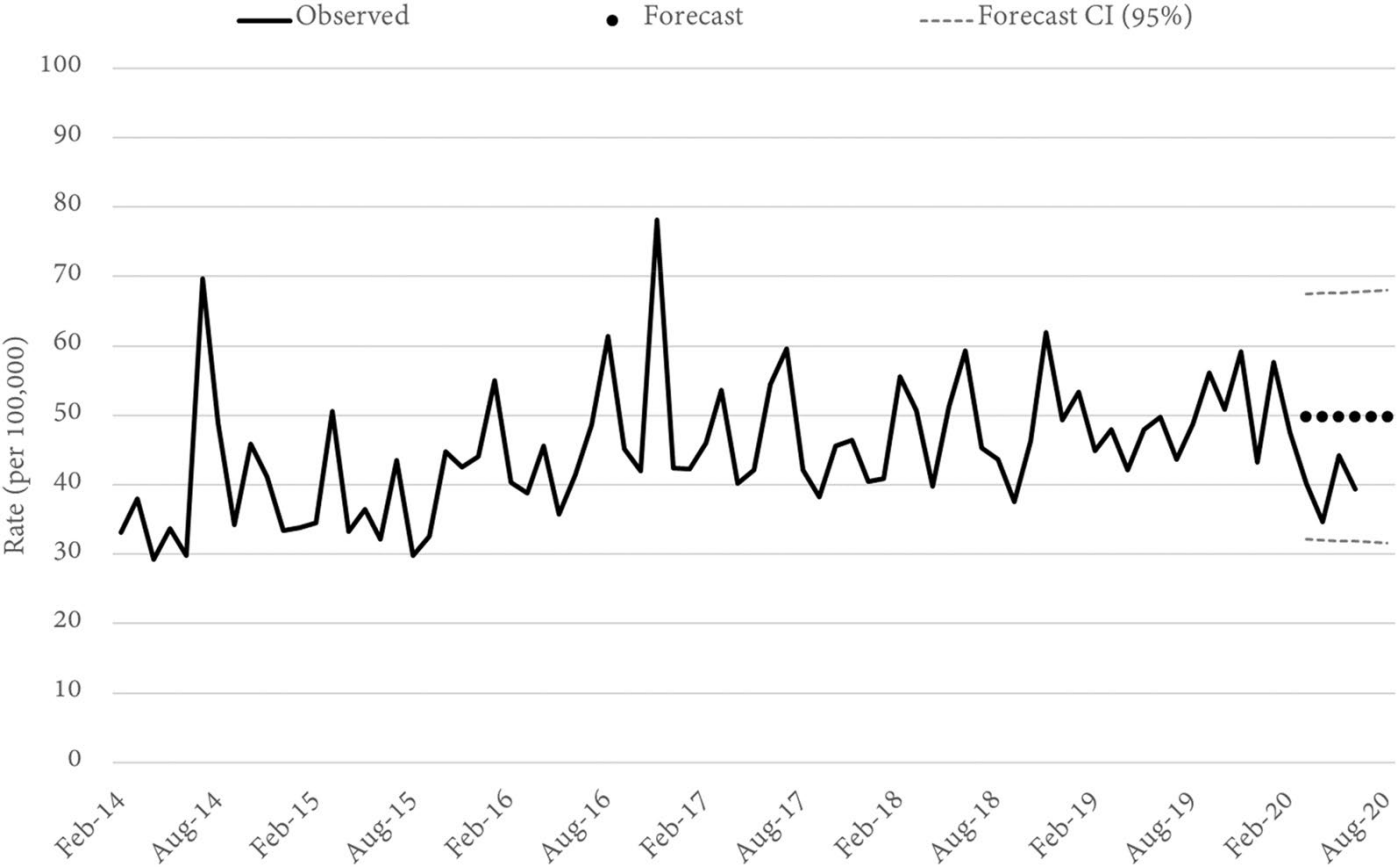
Queensland FRAUD rates and forecasts. Source: Queensland offence rates, Open Data Portal

In April, fraud offences in Queensland were 31% lower than forecast. This was the lowest rate recorded since late 2015, although it did not fall below the lower bound of the 95% confidence interval (Fig. 7). Month-to-month rates of fraud offending are generally low and the time series, both at the LGA and statewide level, were quite volatile. Consequently, only 13 LGA models were able to be estimated with sufficient accuracy and of these models the confidence intervals were relatively wide and the lower-bound often fell below zero. Consequently, it was difficult to reliably assess changes in fraud offending, with one exception—fraud in Cairns (a northern coastal city and tourist location) experienced a 164 percent increase in fraud during June.

### Robbery

**Fig. 8:**
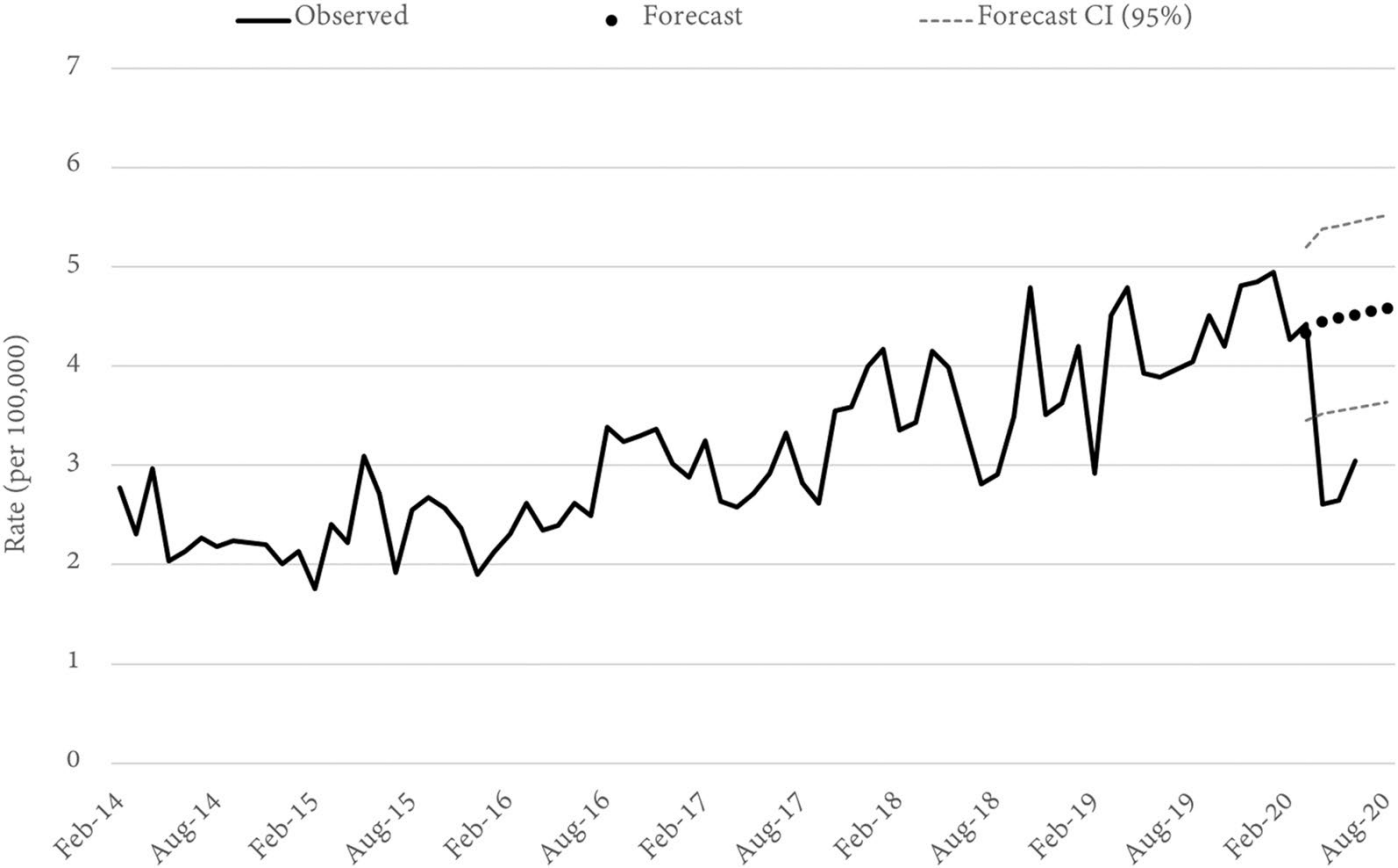
Queensland ROBBERY rates and forecasts. Source: Queensland offence rates, Open Data Portal

Robbery is an acquisitive violent offence, often committed for financial gain and not typically committed against familial relations or close acquaintances for emotive reasons. Most robberies in Australia occur in open-street or community locations (51%) or other retail, industrial or business locations (32%) (Australian Bureau of Statistics 2020e) and thus share similar spatial characteristics to other non-violent forms of property crime. Statewide, robbery rates were unchanged (relative to forecast) in March, then down 41 percent in April and May and down 33% in June (Fig. 8).

Not unlike fraud, robbery offending is relatively uncommon and difficult to forecast at the discrete LGA level. Only eight models were assessed as sufficiently reliable for our analysis. In no case was the rate of robbery in March higher or lower than forecast, while in April, all eight LGAs recorded a decline (six were statistically significant). The same was true for May and June where all eight LGAs were trending lower than forecast.

### Property damage

**Fig. 9:**
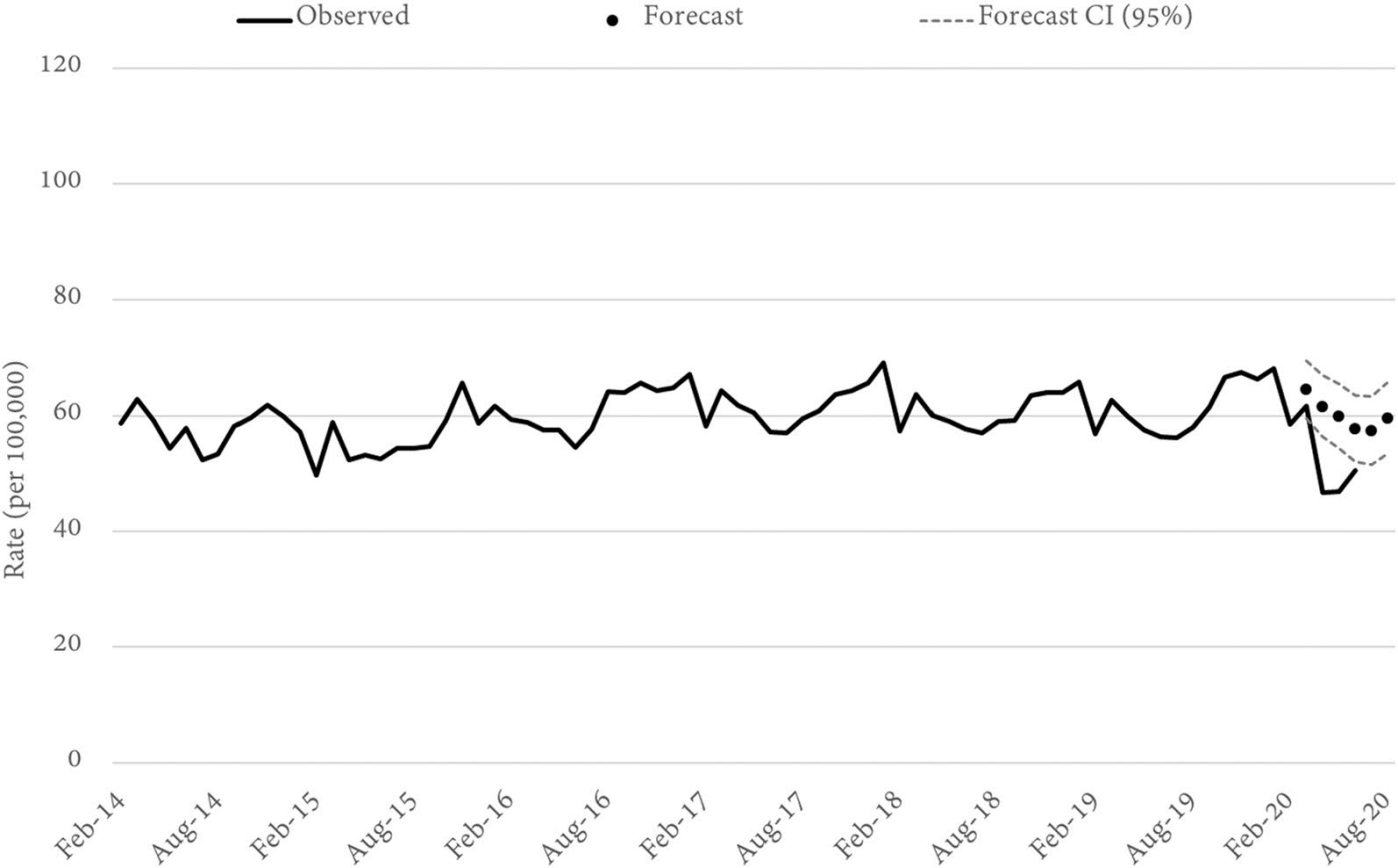
Queensland PROPERTY DAMAGE rates and forecasts. Source: Queensland offence rates, Open Data Portal

Property damage occurs at a rate of approximately 60 offences per 100,000 of the Queensland population. In March, the statewide rate tracked consistent with the forecast. By April, property damage was statistically lower than forecast (down 24%) and remained so through May and June (Fig. 9). Across the state, reliable models could be estimated for 41 LGAs. Of these, 35 recorded a lower-than-forecast rate of property damage in April—ten were statistically significant. Generally, the property damage trends mirror those seen for other property offence types, which is not surprising given that property damage is often an offence that is co-recorded with other forms of theft (such as residential and non-residential burglary). That said, the LGA-specific results show a small number of locations which seem to have had a differential experience during the COVID-19 restrictions. For example, Logan City and Fraser Coast LGAs both experienced a statistically significant increase in property damage during March as social distancing began to take effect. Notably, in the same month both locations also had a significant increase in non-residential (Logan) or residential burglary (Fraser Coast) and Logan also happens to be one of the few locations which saw a minor increase in shop stealing.

Further, at a statewide level property damage appears to have been most impacted in April (down 24%), staying relatively consistent in May and the returning closer to trend in June. This temporal profile was not consistent across Queensland with property damage in the Gold Coast, Ipswich and Townsville, for example, being lowest (relative to forecast) in May. In Redland city, the lowest relative rate was seen in June, although this was still not statistically significant.

## Discussion

This study offers an analysis of the impact of COVID-19 containment measures on recorded property crime rates in Queensland and examined whether the observed impacts varied across the state’s 77 LGAs. We use an iterative univariate ARIMA framework to model the six-year trend in the statewide rates of six property crime types (shop stealing, motor vehicle theft, other theft, burglary, fraud, robbery and property damage) in Queensland, Australia. We capitalize on the period-by-period and seasonal patterns in each series to generate dynamic forecasts (and their 95% confidence intervals) and we compare these forecasts to the observed rates for March through to June 2020. March was the month in which all key social distancing regulations were first brought into force, but not until late in the month. Social distancing was recommended but, for most of March, not mandated. In April, full social distancing restrictions were in place and not relaxed until May 1. At an aggregate statewide level, our results show statistically significant reductions in all property offences types from April and which continued through until June. The one exception was for fraud offending, which declined to a recent-historical low, but not enough to exceed the 95% confidence interval. For the month earlier, when social distancing was recommended, but not mandatory, we found statistically significant declines in shop-stealing, other-theft and residential burglary, and an increase in non-residential burglary.

When viewed through the lens of routine activities and crime pattern theories, these findings are consistent with theoretical expectations. While there was a demonstrable change in mobility towards the end of March, the largest impact on mobility was not observed—and then sustained—until the month of April. Changes in property crime have tended to follow the expected pattern, given the changes to mobility, and are largely consistent with studies conducted overseas and in Australia (Ashby 2020a; Boman and Gallupe 2020; Borrion et al. 2020; Campedelli et al. 2020a, b; Felson et al. 2020; Gerell et al. 2020; Halford et al. 2020; Kim and Leung 2020; Mohler et al. 2020; Rmandic et al. 2020; Rosenfeld and Lopez 2020). In fact, it would appear that the significant reductions in crime observed in Queensland exceeded those observed in the United States, and are higher than those observed in the only other Australian studies to date (Kim and Leung 2020; Rmandic et al. 2020). While in the more recent months of May and June there were some small increases in certain property crime types—shop stealing, other theft, residential burglary and robbery—, crime levels remained well below their forecasted levels. This is despite the staged re-opening of social and business activity which commenced as early as 2 May 2020.

One of the three property crime types signaling an early change was shop stealing and these offences are more likely to occur in high use business districts which normally have significant pedestrian traffic. Consequently, shop theft appears to have declined earlier than most other offence types, most likely due to the statewide social distancing rules which mandated the closure of some retail precincts, limited trading hours of major stores, and which saw an increase in the presence of physical security personnel to monitor social distancing and intervene to prevent panic buying. Each of these actions, made in the interests of public health and safety, are nevertheless likely to have a significant impact on retail-related crime. Specifically, we believe that the opportunities for shop stealing were limited (by restrictions on retail opening hours), target hardened (by the increased presence of surveillance and security), or removed altogether (by retail store closure), meaning that fewer motivated offenders could take advantage of (or encounter) opportunities to steal. This is supported, in part, by Google's mobility trend data, where there was a large but gradual decrease in the use of retail and recreation locations, and an increase in time spent at home, towards the end of March. It is possible that the impact of these containment measures, particularly in terms of influencing routine activities, will have first had their greatest impact on those crime opportunities directly connected to retail locations that were closed, guarded by security, or limited by restrictions in trading hours.

For the category of 'other theft' we offer a similar explanation in that the convergence of motivated offenders with vulnerable victims was likely to have been significantly impacted by the re-configuring of routine activities connected to key retail and economic activities. This category of other theft captures non-assaultive stealing offences in which the primary victim was not a retail premise. This would include the theft of other personal items and belongings, such as mobile phones, handbags and wallets, often in business, retail or entertainment districts, or on public transport. In fact, one Australian study showed as much as 60 percent of these stealing offences occurred on public transport, at workplaces or at licensed premises, all of which have been significantly impacted (Burgess and Grech 2011). A further 25 percent of offences occurred in other public spaces. For the same reasons as above, the opportunities for this type of crime are likely to have been significantly reduced as large segments of the population opted to stay at home and limit the frequency and length of their visits to public places. However motivated some offenders might have been, it is undoubtable that strong social distancing regulations limited the number of vulnerable victims and thus reduced the number of possible criminal opportunities.

The early downward shift in residential burglary for March coincided with a statistically significant increase in non-residential burglary. We suggest that residential burglary declined early, in part because the recommendation of social distancing encouraged many more people in Queensland to stay and work from home, even before the formal restrictions were put into effect. With more people staying and working from home, residential buildings are likely to have benefited from a significant increase in passive surveillance and guardianship. Given this, the complementary rise in non-residential burglary for March might be explained by the same factors. Businesses were closing (or reducing their trading hours) and pedestrian traffic in business or industrial districts was likely to have been much lower than usual making non-residential locations a suitable and potentially attractive alternative to residential thefts. In particular, non-residential burglaries likely also include business such as restaurants and general office spaces, places which have products that are easy to steal and sell on the secondary markets.^10^ By April, both types of burglary were significant below trend. The decline in non-residential burglary is likely to have occurred once businesses and industrial districts were fully closed. Similar patterns in non-residential burglary have been observed elsewhere (Ashby 2020a; Hodgkinson and Andresen 2020), with impacts on burglary shown to depend on the composition of residential and non-residential land use (Felson et al. 2020).

The routine activity and opportunity structures which make possible more serious forms of property crime, namely robbery and motor vehicle theft, were not as easily or quickly influenced by social distancing, with reductions in these two crime types not observed until April. As was expected, however, the increase in time spent by people at home has increased the level of informal guardianship, both by residents and their neighbors, which likely deterred would-be offenders from stealing vehicles parked in driveways, garages and residential car parks in the same way as it reduced opportunities for burglary. Similarly, the restrictions on movement, and resultant decrease in time spent at workplaces, in retail precincts and using public transport prevented vehicles from being parked in places or contexts that make them vulnerable to theft. Of course, the increase in capable guardians, and reduction in suitable targets, likely also coincides with the absence of motivated (potential) offenders, who will have been restricted in their movement by containment measures.

A key objective of this study was to explore the heterogeneity of Queensland’s crime-rate experience when mapped across its 77 LGAs. The fundamental question of interest here is whether the COVID-related crime decline was universal, and where it was not, whether any differences might be equally understood using the same theoretical perspectives. To be sure, Queensland’s 77 LGAs are quite diverse in terms of their population and economic foundations. We might expect, therefore, the impact of COVID-19 in the bustling metropolitan South East (where the Capital, Brisbane, is located) to be different from the western or northern townships which surround the city (Toowoomba, Logan, Redlands, for example), or the agricultural and tourism hubs in the north or far north coast (Sunshine Coast, Mackay, Cairns and Townsville). For theft and related offences the story was mostly of a generalizable and universal impact where there was minor variability around a common trajectory of significant and protracted declines in the rate of offending/detection (at least until June). The variation that did exist appeared consistent with the view that retail and other theft was likely to decline earlier and more rapidly in city LGAs where there are large central business and retail precincts.

For motor vehicle theft, there was no statewide change in March, but underlying this aggregate result were 13 LGAs which recorded an increase, four of which were statistically significant. We note that of the 12 most populated LGAs two recorded a statistically significant increase in March—the Gold Coast and Sunshine Coast Councils—both tourism locations on the northern and southern fringe of the capital city metropolitan region. We note a similar pattern for non-residential burglary which increased significantly in 21 LGAs during March only to reverse into decline from April to June. In the most populous LGAs, these significant increases in non-residential burglary occurred in four locations—the Gold Coast, Sunshine Coast, Logan City and Redland City. Again, two of these locations are suburban/tourism locations, while the others are inner-regional city centers with major manufacturing and industrial business districts. We believe that these increases in March, both for motor vehicle theft and non-residential burglary, were the consequence of some displacement from other crime opportunities.

Further research is needed to understand the reasons for the variation we have uncovered in Queensland (both across LGAs and between crime types in the same LGA). However, given that all regions were equally impacted by the same set of containment measures at the same time (because they were statewide) we believe it is likely that the variability has been strongly influenced by the underlying opportunity structures that exist within each LGA. An equally plausible explanation is that containment measures have the capacity to affect different communities in different ways, perhaps as a result of their different population and socio-economic structures. This, in part, might help to explain why some LGAs experienced less of an overall decline in some crime types, recognizing that social distancing may have prevented some crime, but not as much as might have been the case in more affluent and economically resilient areas.

There are some limitations with our analysis that are important to acknowledge. First, we were reliant on recorded crime data, because they are available much more quickly than other forms of data on crime (e.g., victimization data), and because they permit more fine-grained analysis of changes of overtime and in different locations. While reporting rates are higher for property than violent crime, not all property crime is reported to police. And we do not know how reporting behavior has been influenced by COVID-19. Also, we were limited to monthly data. Other studies have had access to weekly or even daily data; however, these data were not readily available (at least publicly) for the full range of crime types examined in this study. This may mask some of the changes that might have occurred in recorded crime following the introduction of containment measures. As well, while we focused on short-term changes in response to government policy changes, we encourage longer-term analyses. Finally, we focus our work on Queensland, in large part because the data are available for analyses. Other cities (states) in Australia experienced shut-downs, re-openings, and further lockdowns (see Melbourne, Australia in July 2020). Analyses of crime trends in these locations—especially as it is the winter time in the July–August timeframe—would be of interest.

Given the significant disruption to people’s day to day activities, we have drawn heavily on routine activity theory and crime pattern theory to explain these early changes to property crime. We recognize, however, that as containment measures are relaxed, other factors may begin to operate and influence crime rates in different ways. For example, higher rates of unemployment or under-employment have already been observed and are likely to be present in the longer-term, especially among youth and young adult populations, which will likely exert considerable strain on communities. How this will impact crime remains to be seen, although past experience suggests it may result in longer-term changes to the overall pool of motivated offenders, or offender motivations (Phillips and Land 2012). In any case, how property crime rates respond in the longer term to this once-in-a-generation global event will provide invaluable foreground for theoretical analysis into patterns of offending.

## Conclusion

As the novel coronavirus (SARS-cov-2) emerged in the early months of 2020, different countries across the globe responded in a multitude of ways. In Australia, the public health response was comparatively swift and strict, driven by a strong commitment to containment and outbreak mitigation. For this reason, Australia is an important comparative site for the study of COVID-19 and its impact on crime. The north-eastern state of Queensland—where the current study data is drawn-was the first to announce COVID-19 as a public health emergency and one of the last to relax interstate travel restrictions. In this study, we conclude that the stay-at-home orders and social distancing requirements resulted in a significant reduction in all property crimes, with the exception of fraud. We also conclude that the property crime types most affected (retail theft, other theft, and burglary) declined rapidly and by the greatest margins as reduced social mobility limited criminal opportunities to offend. Consistent with this theme, the short-term state-wide increase in non-residential burglary is evidence of the displacement of offending to alternative targets that were more accessible and vulnerable. Although the experience in most LGAs was broadly consistent with the state-wide profile, there were are number of key exceptions. Overall, we conclude that regional differences most likely resulted from differences in local demographic, economic and criminal opportunity structures.

## Data Availability

The data use in the study are publicly available from the Queensland Government Open Data Portal.

## Footnotes

1 It is also worth noting that after the initial spike of COVID-19 cases in Queensland (the highest was 130 cases on the April 4), the state has averaged 1-2 new cases per day—almost all of which were recently returned travellers in quarantine. Unlike elsewhere in Australia (for example, Victoria), Queensland has had almost no community transmission since April and has not experienced a second wave. At the time of writing, there have been only 1,197 cases of COVID-19 in Queensland since the pandemic began (Queensland Department of Health 2020).

2 A very recent study was also published using data from Queensland (see Andresen and Hodgkinson 2020). The methodology of that paper is different to the analyses presented here, although the findings are similar.

3 Although beyond the scope of the data in this study, it is worth noting that on 3 July 2020, Queensland returned to business-as-usual (mostly) with businesses and social activities restricted only insofar as they were required to operate within a COVID-safe plan. Inter-state travel was still prohibited and was only relaxed to some interstate travelers in early November.

4 As developmental and life-course criminologists have long demonstrated (Payne & Piquero, 2020), a substantial proportion of all crime is committed by a small proportion of the population. COVID-19 related strains might not cause a sudden and short-term increase in criminal participation, but it could reasonably trigger an increase in the individual-level offending of those who are already experiencing long-term and high frequency strains.

5 Previous analyses of these data focused exclusively on violent crime (see Payne et al. 2020).

6 Although a longer series of historical data is available for Queensland, we have limited this analysis to crime rates recorded from February 2014. Before that time, there was an observable structural break in the trend, one for which there has not yet been an empirical or theoretical explanation. Modelling the data from 2014 onwards provide both sufficient information for forecasting intent of this paper, but also limits the extent to which the unknown structural break influences the estimation of AR, I and MA parameters.

7 ARIMA models differ from linear OLS models in a number of important ways. The most important is their capacity to model the time series’ moving average. A moving average captures the correlation between lagged errors and is not, therefore, an observed variable capable of estimation in OLS.

8 These four models are ARIMA(0*,d*,0), ARIMA(2,*d*,2), ARIMA(1,*d*,0) and ARIMA(0,*d*,1).

9 Readers should note that for a number of crime types and in a number of LGAs, our decision to limit the analysis to estimable models means that in some cases there are only a small number of LGAs with forecasted outcomes. In the case of fraud, for example, forecasts could be generated for only eight LGAs.

10 In New York City, for example, there were widespread reports of an increase in thefts from shops, restaurants and other businesses, especially at the outset of city-wide lockdowns (Pagones 2020).
